# Chloroquine resistant vivax malaria in a pregnant woman on the western border of Thailand

**DOI:** 10.1186/1475-2875-10-113

**Published:** 2011-05-05

**Authors:** Marcus J Rijken, Machteld E Boel, Bruce Russell, Mallika Imwong, Mara L Leimanis, Aung Pyae Phyo, Atis Muehlenbachs, Niklas Lindegardh, Rose McGready, Laurent Rénia, Georges Snounou, Pratap Singhasivanon, François Nosten

**Affiliations:** 1 Shoklo Malaria Research Unit, PO Box 46 Mae Sot, Tak 63110, Thailand; 2 Laboratory of Malaria Immunobiology, Singapore Immunology Network, Biopolis, Agency for Science Technology and Research (A*STAR), Singapore; 3 Department of Molecular Tropical Medicine and Genetics, Faculty of Tropical Medicine, Mahidol University, Bangkok 10400, Thailand; 4 University of Washington, 1959 NE Pacific Street, Seattle, WA 98195, USA; 5 Faculty of Tropical Medicine, Mahidol University, Bangkok 10400, Thailand; 6 Centre for Tropical Medicine, Nuffield Department of Clinical Medicine, University of Oxford, CCVTM, Oxford OX3 7LJ, UK; 7 INSERM UMR S-945, F-75013 Paris, France; 8 Université Pierre & Marie Curie, Faculté de Médecine Pitié-Salpêtrière, F-75013 Paris, France

## Abstract

Chloroquine (CQ) resistant vivax malaria is spreading. In this case, *Plasmodium vivax *infections during pregnancy and in the postpartum period were not satisfactorily cleared by CQ, despite adequate drug concentrations. A growth restricted infant was delivered. Poor susceptibility to CQ was confirmed *in-vitro *and molecular genotyping was strongly suggestive of true recrudescence of *P. vivax*. This is the first clinically and laboratory confirmed case of two high-grade CQ resistant vivax parasite strains from Thailand.

## Background

Chloroquine (CQ) remains the recommended first-line treatment for *Plasmodium vivax *globally except for Indonesia, Papua New Guinea, the Solomon Islands and Vanuatu where widespread CQ resistance prompted a change in treatment policy [1,2]. Clinical monitoring of CQ efficacy is confounded by relapses derived from the activation of hypnozoites (dormant hepatic forms characteristic of *P. vivax*), making it difficult to categorize post-treatment episodes as recrudescences, re-infections or relapses [3]. Moreover measurement of the intrinsic sensitivity to CQ has been hampered by the difficulty to maintain *P. vivax *in culture. Nonetheless cases of CQ-resistant *P. vivax *have been reported from all continents where malaria is endemic, but never in pregnancy [1]. Previous clinical studies in the non pregnant Thai population where CQ was combined with primaquine did not show cases of highly suspect CQ resistant vivax [4-6]. Combined clinical and laboratory data from a closely monitored Karen pregnant woman on the western border of Thailand highly indicative of CQ resistance by molecular genotyping is presented in this report.

## Case presentation

A 38 year-old pregnant Karen woman (blood group B, G6PD level normal and HIV negative) in her third pregnancy registered at a gestational age of 20^+5 ^weeks (confirmed by abdominal ultrasound) [7]. She lived and worked in the forests on the Thai-Myanmar border and provided written informed consent to participate in a "postpartum susceptibility to malaria" study approved by the Ethics Committees of Oxford University (OxTREC (002_007) and Mahidol University (MUTM 2007-023) which included repeated blood sampling and publication of any data. She gave birth to a growth restricted live born singleton boy without congenital abnormality of 2,540 (±10) grams at a gestational age of 41^+1 ^weeks (<10^th ^percentile) on 19 December 2008. On the day of registration (D0) she presented with fever and was diagnosed with *P. vivax *malaria on the presence of asexual forms in peripheral blood (parasitaemia 9294/μL). Treatment was with CQ (25 mg base/kg total dose, Government Pharmaceutical Organization, Thailand) as per standard protocol. Over the following 227 days she was treated eight times for recurrent *P. vivax *parasitaemic episodes.

## Methods

Parasite species diagnosis of each episode was determined by microscopic examination of Giemsa-stained blood films. This was later confirmed by nested PCR [8] for the episodes that occurred after D21, when suspicion about CQ resistant vivax was raised. *Plasmodium vivax *parasites were genotyped for three polymorphic markers: defined polymorphic regions in the genes encoding for the circumsporozoite surface protein (*Pvcs*), the merozoite surface protein 1 (*Pvmsp1*) or the merozoite surface protein 3α (*Pvmsp3-α*) [3,9,10] and for point mutations or copy number variation in the multi drug resistance gene 1, *Pvmdr1 *[11]. Blood samples were also obtained from the placenta (including by mechanical extraction), umbilical cord and from the infant. Histopathologic analysis of a placenta biopsy was performed on Giemsa-stained cryo-sections.

The intrinsic *ex vivo *sensitivity assays were carried out for chloroquine, artesunate, piperaquine, mefloquine and amodiaquine on a leukocyte-depleted *P. vivax *isolate [12]. The pre-dosed plates employed for these assays were quality assured using a *Plasmodium falciparum *cloned line (PfK1). Dose response curves and IC_50 _(50% inhibitory concentration) values were calculated by fitting the data to a sigmoidal inhibitory E-max pharmacodynamic model using WINNONLIN Ver 4.1 (Pharsight Corporation). The assays were duplicated and the data confirmed independently by three experienced microscopists.

Serum CQ and desethylchloroquine (DECQ) concentrations were measured using solid-phase extraction and liquid chromatography coupled with UV-detection [13].

## Results

**Figure 1:**
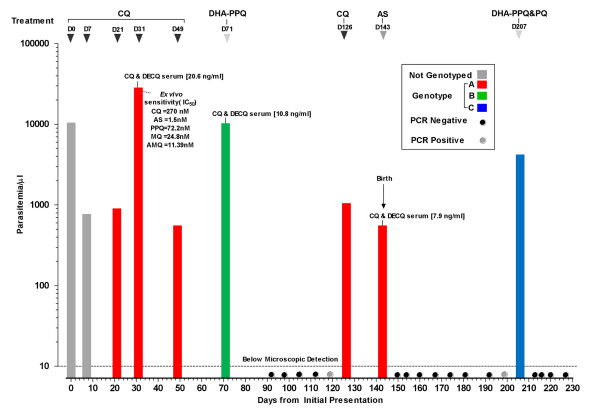
***Plasmodium vivax *infections in a Karen woman observed over 227 days pregnancy and postpartum period**. The *P. vivax *genotypes (indicated by bars of different colours) are based on polymorphisms in 4 genes (*Pvcs*, *Pvmsp1*, *Pvmsp3-α*, and *Pvmdr1*). The treatments administered are indicated on top. PCR spots are indicated with black (negative) and grey (positive) dots. The intrinsic sensitivity profile of *P. vivax *isolate to a range of standard antimalarials (chloroquine (QC), artesunate (AS), piperaquine (PPQ), mefloquine (MQ) and amodiaquine (AMQ)) is shown for the parasites collected on D31. The *in vivo *serum concentrations of CQ + desethylchloroquine (DECQ) are indicated for the samples collected on D31, D71 and D143.

Over the course of 227 days this woman was seen 26 times (see Figure 1). The parasitological and laboratory findings for the samples collected from the pregnant woman are summarized in Figure 1. The persistence of *P. vivax *(parasite count 750/μl, non-symptomatic) on D7 post CQ administration prompted a second CQ course but parasites were still present in the blood on her next visit (D21) (parasite count 850/μl, non-symptomatic). Chloroquine was administered again (3^rd ^course). Symptoms and a higher parasite density (parasite count 27500/μL) were present on D31, and a complete supervised course of CQ was given. An asymptomatic *P. vivax *parasitaemia on D49 (parasite count 500/μL) pressed the administration of a fifth course of CQ. When vivax parasites re-appeared on D71 (parasite count 10,000/μL, symptomatic) the women was given a course of dihydroartemisinin (DHA) - piperaquine (PPQ), (6.75 mg/kg DHA and 54 mg/kg PPQ, 3 days, Holley Pharm, Peoples Republic China), the most effective treatment against uncomplicated vivax malaria in West Papua[14], and parasites were undetectable by microscopy over the next five visits (D92-D119). A parasitaemic episode on D126 (parasite count 1000/μL, non-symptomatic) close to the expected delivery date was treated with CQ. But on delivery (D143) parasites were still detected in the mother's peripheral blood (parasite count 500/μl, non-symptomatic) and placenta (detected by PCR of placental blood). Placenta histologic findings were subtle: rare parasites, very mild inflammatory infiltrate and scant pigment deposition in the intervillous space. This infection was treated with artesunate (AS) (2 mg/kg/day, 7 days, Guilin, PRC) and parasites were not detected in blood smears taken on subsequent visits (D150 - D199), but on D206, 63 days postpartum an asymptomatic infection (parasite count 4,000/μl) was detected again. This was treated with DHA-PPQ and primaquine (PQ) (22.5 mg base/day for 14 days, GPO, Bangkok, Thailand). The parasites were promptly cleared and the blood smear remained negative until the end of the follow-up which was three months post-partum (D227). There were no parasites found in the cord blood or in the infant, who remained negative thereafter.

Combined CQ + DECQ serum concentrations obtained at three of the clinical episodes (D31 = 20.6, D71 = 10.8 and D143 = 7.9 ng/ml) indicated adequate drug exposure and were around the 15 ng/ml serum threshold considered therapeutic against CQ-susceptible parasites[15]. Blood samples were also collected on these days for *ex vivo *sensitivity assays but only one (D31) harboured parasites at a developmental stage suitable for meaningful analysis [12]. The *ex vivo *susceptibility profile clearly indicated *P. vivax *with reduced sensitivity to CQ (IC_50 _= 270 nM) though not to the other anti-malarials tested (Figure 1).

PCR analysis confirmed the microscopic diagnoses and interestingly two additional sub-microscopic infections (D119, D199) were observed the week before they were detected microscopically. Genotyping revealed that the patient was successively infected by three distinct *P. vivax *populations (Figure 1 and Table 1). Parasites from D21-D49 were of similar genotype to those of D126-D143 but differed from the two distinct populations present on D71 and D206. All parasites had a single copy of *Pvmdr1 *and a T958M mutation but for those from D21-D49 and D126-D143 an additional Y976F mutation was observed.

**Table 1:** Molecular criteria for the classification of three genotypes observed in the malaria patient.

Genetic Locus	Genotype A	Genotype B	Genotype C
Pvcs*			

Size (bp)	650	650	650

*Alu *I (VK210-type)	D	D	D

*Bst *NI (VK247-type)	U	U	U

*Scr *FI/*Bbs *I	U/U	D/U	U/U

(VK210)			

Type	VK210 (i)	VK210 (ii)	VK210 (i)

*Pvmsp1*			

F1 fragment (bp)	400	400	400

Pvmsp3α*			

*Alu *I (bp)	560, 260, 190, 170	560, 460, 200, 180, 150, 120	560, 460, 200, 180, 150, 120

*Hha *l (bp)	1100, 460, 260, 180	1100, 440, 260, 220	1100, 440, 260, 220

Type	(i)	(ii)	(ii)

*Pvmdr1 *=			

T958M	M	M	M

Y976F	F	F	Y

F1076L	L	L	F

Copy Number	1	1	1

Type	(i)	(i)	(ii)

* Following incubation with specific restriction enzyme the amplified *Pvcs *fragment is either digested (D) or remains uncut (U). *Alu *I and *Bst *NI digestion indicate whether the central polymorphic repeat region, which is amplified for the genotyping, is of the VK210 or VK247 type, while *Scr *FI and *Bbs*I digestion indicate the type of pre- and post-repeat pattern surrounding the VK210 repeat type allelic variants.

With respect to *Pvmsp3α*, the pattern of fragments obtained following restriction enzyme digestion provides a means to distinguish between different parasite genotypes. Different allelic variants are denoted with (i) and (ii).

= The amino acid residue encoded is provided, as is the average copy number. Different allelic variants are denoted with (i) and (ii).

## Conclusion

This woman had multiple *P. vivax *episodes over a 227-day period during pregnancy and post-partum, characterized by repeated recurrences of *P. vivax *parasites following CQ treatment and resulting in a growth restricted neonate. The *in vivo *observations of *P. vivax *recurrences were associated to an *ex vivo *drug sensitivity assay showing a CQ IC_50 _of 270 nM. This value is indicative of high-grade CQ vivax resistance as it exceeds recently published IC_50 _medians for CQ of 37 nM for Thai (CQ sensitive) and 114 nM for Papuan (CQ resistant) isolates [12]. Indeed on two occasions (D31 and D71) each with a different genotype the parasitaemia increased substantially despite the presence of therapeutically adequate drug concentrations. The combined CQ+DECQ concentrations were similar to what has been reported for resistant vivax cases in a study in Myanmar [16]. It is interesting that the *Pvmdr1 *Y967F mutation was found in the parasites from two genotypically distinct episodes (Genotypes A and B). This putative marker of CQ resistant vivax was relatively rare (only 20%) in isolates from the Thai-Myanmar border [17], but was present in 97% of the isolates from West Papua where CQ vivax resistance is widespread[11]. This could imply that there are at least two strains of chloroquine resistant parasites circulating in this area.

The CQ-resistant *P. vivax *in this Karen woman might have been acquired in Myanmar where she worked in the forests during her pregnancy, but she could have also acquired the infection in Thailand where she resides. Irrespective of its origin the fact that CQ-resistant vivax malaria appears to be spreading is a matter for concern.

Evidence based treatment guidelines for CQ resistant vivax infections are urgently needed to prevent repeated dosing of an ineffective drug, a practice known to enhance the selection and spread of resistant parasites. However, the choice of safe and effective drugs to replace chloroquine is very limited. Some artemisinin-combinations, such as DHA-PPQ or artesunate-mefloquine, are effective against *P. vivax *[1], but artemisinins are contraindicated in the first trimester and primaquine, the sole drug effective against hypnozoites, is also contraindicated in pregnant women. In South East Asia, *P. vivax *is resistant to sulphadoxine-pyrimethamine [18], a drug considered to be safe during pregnancy. Careful monitoring of the efficacy of CQ in the treatment of *P. vivax *in this region is needed especially in pregnant women to reduce the harmful effects on mothers and infants [19].

## Consent

Written informed consent was obtained from the patient for publication of this case report and any accompanying images. A copy of the written consent is available for review by the Editor-in-Chief of this journal.

## Competing interests

The authors declare that they have no competing interests.

## Authors' contributions

MJR, MEB, APP, and RMG were responsible for clinical work, design of the study, initiated and drafted the manuscript. BR and MLL did the microscopic examination, nested PCR and sensitivity assays. MI carried out the genotyping and drafted the manuscript. AM carried out the histopathology examination and drafted the manuscript. NL carried out the measurements of drug concentrations and drafted the manuscript. MJR, MEB, BR and GS combined all laboratory and clinical results into the manuscript. LR, GS, PS and FN participated in the design of the study, provided decisive comments and finalized the manuscript

All authors read and approved the final manuscript.

## References

[B1] Baird JK Resistance to therapies for infection by *Plasmodium vivax* Clin Microbiol Rev 2009 22 508 534 10.1128/CMR.00008-09 19597012 PMC2708388

[B2] Harijanto PN Malaria treatment by using artemisinin in Indonesia Acta Med Indones 2010 42 51 56 20305333

[B3] Imwong M Snounou G Pukrittayakamee S Tanomsing N Kim JR Nandy A Guthmann J-P Nosten F Carlton JM-R Looareesuwan S Nair S Sudimack D Day NP Anderson TJ White NJ Relapses of *Plasmodium vivax *infection usually result from activation of heterologous hypnozoites J Infect Dis 2007 195 927 933 10.1086/512241 17330781

[B4] Congpuong K Na-Bangchang K Thimasarn K Tasanor U Wernsdorfer WH Sensitivity of *Plasmodium vivax *to chloroquine in Sa Kaeo Province, Thailand Acta Trop 2002 83 117 121 10.1016/S0001-706X(02)00090-6 12088852

[B5] Muhamad P Ruengweerayut R Chacharoenkul W Rungsihirunrat K Na-Bangchang K Monitoring of clinical efficacy and in vitro sensitivity of *Plasmodium vivax *to chloroquine in area along Thai Myanmar border during 2009-2010 Malar J 2011 10 44 10.1186/1475-2875-10-44 21324161 PMC3055225

[B6] Tasanor O Ruengweerayut R Sirichaisinthop J Congpuong K Wernsdorfer WH Na-Bangchang K Clinical-parasitological response and in-vitro sensitivity of *Plasmodium vivax *to chloroquine and quinine on the western border of Thailand Trans R Soc Trop Med Hyg 2006 100 410 418 10.1016/j.trstmh.2005.04.024 16497347

[B7] Rijken MJ Lee SJ Boel ME Papageorghiou AT Visser GH Dwell SL Kennedy SH Singhasivanon P White NJ Nosten F McGready R Obstetric ultrasound scanning by local health workers in a refugee camp on the Thai-Burmese border Ultrasound Obstet Gynecol 2009 34 395 403 10.1002/uog.7350 19790099 PMC3438883

[B8] Snounou G Viriyakosol S Zhu XP Jarra W Pinheiro L Do Rosário VE Thaithong S Brown KN High sensitivity of detection of human malaria parasites by the use of nested polymerase chain reaction Mol Biochem Parasitol 1993 61 315 320 10.1016/0166-6851(93)90077-B 8264734

[B9] Bruce MC Galinski MR Barnwell JW Snounou G Day KP Polymorphism at the merozoite surface protein-3a locus of *Plasmodium vivax*: global and local diversity Am J Trop Med Hyg 1999 61 518 525 10548283 10.4269/ajtmh.1999.61.518

[B10] Imwong M Pukrittayakamee S Grüner AC Rénia L Letourneur F Looareesuwan S White NJ Snounou G Practical PCR genotyping protocols for *Plasmodium vivax *using *Pvcs *and *Pvmsp1* Malar J 2005 4 20 10.1186/1475-2875-4-20 15854233 PMC1131918

[B11] Suwanarusk R Chavchich M Russell B Jaidee A Chalfein F Barends M Prasetyorini B Kenangalem E Piera KA Lek-Uthai U Anstey NM Tjitra E Nosten F Cheng Q Price RN Amplification of *pvmdr1 *associated with multidrug-resistant *Plasmodium vivax* J Infect Dis 2008 198 1558 1564 10.1086/592451 18808339 PMC4337975

[B12] Russell B Chalfein F Prasetyorini B Kenangalem E Piera KA Suwanarusk R Brockman A Prayoga P Sugiarto P Cheng Q Tjitra E Anstey NM Price RN Determinants of *in vitro *drug susceptibility testing of *Plasmodium vivax* Antimicrob Agents Chemother 2008 52 1040 1045 10.1128/AAC.01334-07 18180357 PMC2258486

[B13] Blessborn D Neamin G Bergqvist Y Lindegårdh N A new approach to evaluate stability of amodiaquine and its metabolite in blood and plasma J Pharm Biomed Anal 2006 41 207 212 10.1016/j.jpba.2005.10.018 16307860

[B14] Hasugian AR Purba HLE Kenangalem E Wuwung RM Ebsworth EP Maristela R Penttinen PMP Laihad F Anstey NM Tjitra E Price RN Dihydroartemisinin-piperaquine versus artesunate-amodiaquine: superior efficacy and posttreatment prophylaxis against multidrug-resistant *Plasmodium falciparum *and *Plasmodium vivax *malaria Clin Infect Dis 2007 44 1067 1074 10.1086/512677 17366451 PMC2532501

[B15] Baird JK Basri H Bangs MJ Subianto B Patchen LC Hoffman SL Resistance to chloroquine by *Plasmodium vivax *in Irian Jaya, Indonesia The Am J Trop Med Hyg 1991 44 547 552 10.4269/ajtmh.1991.44.547 1676566

[B16] Guthmann J-P Pittet A Lesage A Imwong M Lindegårdh N Myo Min Lwin Than Zaw Annerberg A De Radiguès X Nosten F *Plasmodium vivax *resistance to chloroquine in Dawei, southern Myanmar Trop Med Int Health 2008 13 91 98 10.1111/j.1365-3156.2007.01978.x 18291007

[B17] Imwong M Nair S Pukrittayakamee S Sudimack D Williams JT Mayxay M Newton PN Kim JR Nandy A Osorio LE Carlton JM White NJ Day NP Anderson TJ Contrasting genetic structure in *Plasmodium vivax *populations from Asia and South America Int J Parasitol 2007 37 1013 1022 10.1016/j.ijpara.2007.02.010 17442318

[B18] Imwong M Pukrittakayamee S Looareesuwan S Pasvol G Poirreiz J White NJ Snounou G Association of genetic mutations in *Plasmodium vivax *dhfr with resistance to sulfadoxine-pyrimethamine: geographical and clinical correlates Antimicrob Agents Chemother 2001 45 3122 3127 10.1128/AAC.45.11.3122-3127.2001 11600366 PMC90792

[B19] Nosten F McGready R Simpson JA Lay Thwai Kyaw Balkan S Cho Thein Hkirijaroen L Looareesuwan S White NJ Effects of *Plasmodium vivax *malaria in pregnancy Lancet 1999 354 546 549 10.1016/S0140-6736(98)09247-2 10470698

